# Clinical Outcomes and Failure Rate of Triangular Fibrocartilage Complex Foveal Repair Were Comparable between Arthroscopic and Open Techniques

**DOI:** 10.3390/jcm13102766

**Published:** 2024-05-08

**Authors:** Shin Woo Lee, Jung Jun Hong, Seung-Yong Sung, Tae-Hoon Park, Ji-Sup Kim

**Affiliations:** 1 Department of Orthopaedic Surgery, College of Medicine, Ewha Womans University Seoul Hospital, Seoul 07804, Republic of Korea; periodics@nate.com; 2 Department of Orthopaedic Surgery, Yonsei Wa Hospital, Incheon 21557, Republic of Korea; ultrah03@naver.com; 3 Department of Orthopaedic Surgery, College of Medicine, Catholic-Kwandong University, Incheon 22711, Republic of Korea; sysung@ish.ac.kr; 4 Department of Orthopaedic Surgery, Gangnam Nanoori Hospital, Seoul 06048, Republic of Korea; thpark2190@naver.com

**Keywords:** arthroscopy, triangular fibrocartilage complex(TFCC), foveal repair

## Abstract

**Background:** This study compared clinical outcomes between arthroscopic and open repair of triangular fibrocartilage complex (TFCC) foveal tears in chronic distal radioulnar joint (DRUJ) instability patients. **Methods:** A total of 79 patients who had gone through foveal repair of TFCC using arthroscopic technique (n = 35) or open technique (n = 44) between 2016 and 2020 were retrospectively analyzed. The visual analog scale (VAS) score for pain, active range of motion (ROM), grip strength, Mayo Modified Wrist Score (MMWS), Disabilities of the Arm, Shoulder, and Hand (DASH) questionnaire score, and Patient-Rated Wrist Evaluation (PRWE) score at 2-4-6-12-24 months postoperatively were compared between two groups. **Results:** Two years after the operation, clinical parameters (VAS, MMWS, DASH, and PRWE), grip strength, and ROM showed significant advancement in the two groups in comparison to their values measured preoperatively (*p* < 0.001). Nonetheless, we could not identify any statistically significant differences in the above clinical factors between the two groups. The arthroscopic group showed a better flexion–extension arc at 2 months and supination–pronation arc at 2 and 4 months than the open group (*p* < 0.001). There were no significant differences between the two groups at 2 years postoperatively. Ten patients (12.6%) had recurrent instability (three in the arthroscopic group and seven in the open group, *p* = 0.499). Similarly, both groups showed no significant difference in the return to work period. **Conclusions:** Arthroscopic foveal repair of TFCC provided similarly favorable outcomes and early recovery of pain and ROM compared to open repair.

## 1. Introduction

TFCC injuries are increasingly acknowledged for the reason of ulnar aspect wrist pain. They could result from acute trauma [1,2,3] or athletic activity, such as racquet sports, baseball, and contact sports [4,5,6]. Considerable long-term disability might arise, and if these injuries go unnoticed, they can lead to inadequate treatment and a lack of suitable rehabilitation [6].

The triangular fibrocartilage complex (TFCC) contributes significantly to stabilizing the distal radioulnar joint (DRUJ) [1,7,8,9,10]. TFCC injury could be the cause of chronic ulnar-side wrist pain and instability of DRUJ, especially if accompanied by a proximal detachment of TFCC at the fovea of the ulnar head (Atzei classification class II or III TFCC tear) [11,12].

In DRUJ instability, successful outcomes were reported for foveal reattachment using open and arthroscopic techniques [13,14,15,16,17]. A biomechanical study [18] showed that the arthroscopic repair technique demonstrated superior results in strength compared to the open repair technique. Despite the excellent biomechanics of the arthroscopy technique over the open technique for TFCC foveal repair, there has been debate on clinical outcomes. To date, research analyzing the clinical consequences and complications of arthroscopic and open repair techniques for TFCC foveal injury is lacking [7,19].

Thus, we aimed to compare surgical outcomes and complication rates between arthroscopy and open repair techniques in patients with Atzei classification II or III isolated TFCC tears by performing periodic measurements of the visual analog scale (VAS) score for pain and time-dependent clinical patient-reported outcomes utilizing questionnaires.

We developed a hypothesis suggesting that arthroscopic TFCC foveal repair could yield superior clinical results and could lead to faster recovery compared to the open repair technique.

## 2. Materials and Methods Study Population

The present study achieved approval by the institutional review board (IRB) of Catholic Kwandong University, and consent from the participants was exempted on account of the retrospective nature. The authors went through an evaluation of the electronic medical records of 131 patients retrospectively who had gone through either arthroscopic or open repair for TFCC foveal tears operated by a single orthopaedic surgeon between January 2016 and August 2020. We assigned patients to an arthroscopy or open group according to the surgical procedure that they underwent (arthroscopy-assisted repair or open repair, respectively). The patient was not assigned with randomization, and open foveal repair was operated on earlier during the study (January 2016 to December 2018); in contrast, arthroscopic foveal repair was carried out later on (January 2019 to August 2020). The indication of TFCC repair was pain and instability that had hindering effects on activities of daily living, which showed no response to conservative treatments for a minimum of 3 months. All patients showed positive foveal signs and instability in the ballottement test. We made a confirmative diagnosis of tears in the fovea of TFCC by positive hook test in every patient, as demonstrated during diagnostic arthroscopy [20,21]. The patient exclusion was conducted by the following criteria: <2 years of follow-up duration (five patients in the arthroscopy group and seven in the open group), previous fracture or operation history of the affected limb (four in the arthroscopy group and eight in the open group), accompanying carpal ligament injury (nine in the arthroscopy group and eleven in the open group), insufficient medical records (six patients), and laborers’ compensation claim (two patients). None of the patients revealed articular cartilage degeneration or arthritic changes in the radiocarpal and distal radioulnar joints. Ultimately, 79 patients (35 in the arthroscopy group and 44 in the open group) who conformed to the inclusion criteria were involved, with all patients following up using a subjective questionnaire at least 2 years subsequent to the operation during outpatient service.

### 2.1. Surgical Technique

The patient underwent wrist arthroscopy under general anesthesia in the supine position. A total of 250 mmHg pneumatic tourniquets were applied to the upper limb of the affected side. A total of 1 g of Flomoxef (2nd generation cephalosporin) prophylactic antibiotics were used. Utilizing a standard 3/4 wrist joint portal, diagnostic arthroscopy was performed to confirm TFCC foveal tears and other pathologic lesions inside the wrist joint. The hook probe was introduced via the 6R portal, and we performed trampoline and hook tests [22] to evaluate the tension of the TFCC at its insertion. For postoperative pain control, patient-controlled analgesics, such as Tramadol and NSAIDs, were administered as requested by the patient.

### 2.2. Arthroscopic Repair of the Fovea of TFCC

Once we confirmed the detachment of the fovea of the TFCC, we made a 1 cm size longitudinal incision on the shaft of the ulna near the styloid tip of the ulna. Alongside lifting the periosteum, we drilled two small holes parallel to each other in the ulnar cortex using 1.5 mm Kirschner wires. Following the drilling procedure, we guided Kirschner wires distally to the TFCC foveal footprint using arthroscope assistance. A 2-0 FiberWire (Arthrex, Naples, FL, USA) had been threaded past the bone tunnel and fovea of TFCC using an 18-gauge spinal needle. A 3-0 polydioxanone suture (Ethicon Inc., Somerville, NJ, USA) was looped, advanced past another bone tunnel, and retrieved to obtain the primary suture in the wrist joint. The primary ligature of the suture was applied past the TFCC and extended outward to the bony cortex of the distal ulna. Once the traction device was removed, the suture was securely tied to a 90° flexed elbow and a neutrally positioned forearm. See Video S1 (online).

### 2.3. Open Foveal Repair of the TFCC

After diagnostic arthroscopy, a 4 cm 6-R portal-centered curved incision was created on the dorso-ulnar aspect of the wrist while maintaining vertical wrist traction. If the patient showed subluxation and chronic synovitis of the extensor carpi ulnaris (ECU) tendon during preoperative assessment, we elevated the extensor retinaculum flap over the radius for joint capsule exposure. A joint capsule opening was carefully created through transverse capsulotomy, avoiding any damage to the periphery of the TFCC, to visualize the fovea; the scar tissue was debrided, and the fovea was curetted to prepare well-vascularized cortical bone. An anchor with a four-strand suture (BioComposite SutureTak, Arthrex Med. Inst. GmbH, Karlsfeld, Germany) was placed at the fovea. After the removal of finger traps and traction, the elbow was flexed at 90° with the forearm neutrally positioned; finally, multiple sutures were securely tied around the periphery of the repaired TFCC. The dorsal capsule and retinaculum were closed with 4-0 Vicryl (Ethicon Inc., Somerville, NJ, USA). The retinal tongue was passed deep to the ECU tendon and attached at a more distal retinal site to prevent instability of the ECU. See Video S2 (online).

### 2.4. Postoperative Care

Following the operation, the authors applied a long-arm splint to patients for 4 weeks postoperatively. Then, the rehabilitation program was initiated. We initiated hand therapy involving an active and passive range of motion enhancement program encouraging supination and pronation motion with no resisting force. Patients were advised to perform muscle reinforcement activities resisting external force at postoperative 3 months. We allowed sporting activities 6 months after surgery.

### 2.5. Functional and Clinical Assessments

We obtained subjective and functional results in a prospective manner from two separate experienced orthopaedists who did not participate in the operation or postoperative treatment process, utilizing a variety of implements. Functional indices used are as follows: VAS for resting pain and under stress, active range of motion of the radiocarpal joint (ROM), grip strength, MMWS [23], DASH [24], and PRWE [25] questionnaire scores. We measured at 2, 4, 6, 12, and 24 months postoperatively. Ballottement test was conducted to evaluate DRUJ stability, which was rated as follows: grade 3, overall instability with no endpoint in both dorsal and palmar aspects; 2, instability with no clear endpoint in either dorsal and palmar aspects; and 1, instability of the injured side exceeding that of the normal side accompanying firm endpoints [26]. A hand-operated goniometer was used to measure the ROM of the radiocarpal joint, including pronation–supination, flexion–extension, and radial–ulnar deviation. We assessed grip power utilizing the Jamar Dynamometer (Asimov Engineering, Los Angeles, CA, USA) and expressed it in kg. Preoperative radiologic assessments included posterior–anterior and lateral X-ray images, in addition to the baseline magnetic resonance imaging study, which is the most valuable imaging study for the diagnosis of TFCC tear [27]. Radiographs were obtained during the follow-up. Data were obtained from our electronic medical records. We confirmed the return-to-work period in weeks, according to the patients’ medical records. We also assessed all patients for any surgery-associated (recurrent TFCC rupture, neural injury, infection, joint stiffness, etc.) complications during the entire follow-up duration.

### 2.6. Statistical Analysis

We described continuous variables in terms of mean ± standard deviation after Shapiro–Wilk normality testing. We characterized discrete variables by ratio or frequency. When comparing two groups at a separate postoperative timeline, we used the Student’s *t*-test (or Wilcoxon rank sum test) for the analysis of continuous variables, whereas the chi-squared (in other words, Fisher’s exact) test was utilized to analyze discrete variables. We regarded a *p*-value below 0.05 as indicative of statistical significance. We used statistical software R (version 3.5.1, R Foundation for Statistical Computing, Vienna, Austria) for the entire statistical assessment.

## 3. Results

### 3.1. Patient’s Characteristics

**Figure 1:**
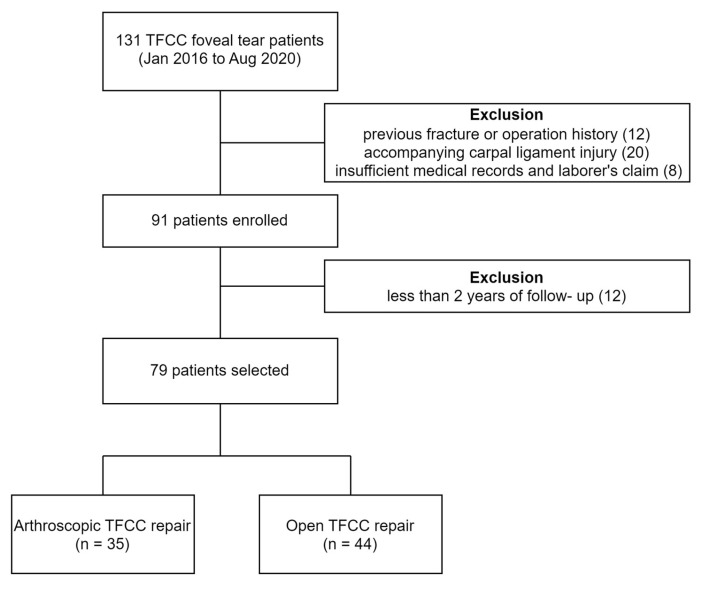
Flowchart of patients assigned to arthroscopic or open TFCC repair.

A total of 79 patients were involved in the present research; 35 had arthroscopic TFCC repair (which was classified as the arthroscopy group), and 44 had open TFCC repair (which was classified as an open group) (Figure 1). The average ages of patients at the time of operation were 40.4 and 39.0 years in the arthroscopy and open groups, and the average symptomatic period until the operation was 15.7 and 11.7 months, respectively. The two groups showed no significant difference in the following factors: age, gender, affected side, smoking history, and time to surgery (Table 1).

### 3.2. Arthroscope Findings and Concomitant Procedures

Ulnar impaction syndrome, defined as central TFCC perforation, was found in 31% (11 of 35) and 30% (12 of 44) of all participants in the arthroscopy and open groups in corresponding order. As for the incidence of ulnar impaction syndrome, no notable distinction was found between the arthroscopy and open groups (31.4% vs. 27.3%; *p* = 0.877). As for the arthroscopy group, arthroscopic debridement of TFCC and wafer procedures (distal ulnar resection) were performed in eight patients, and three patients underwent ulnar shortening osteotomies, whereas for the open group, these procedures were performed on eight and four patients separately. Surgical indication of the wafer procedure was performed when ulnar variance was lower than 3 mm, whereas ulnar shortening osteotomy was performed when ulnar variance was greater than 3 mm [28,29].

### 3.3. Functional and Clinical Outcomes

**Figure 2:**
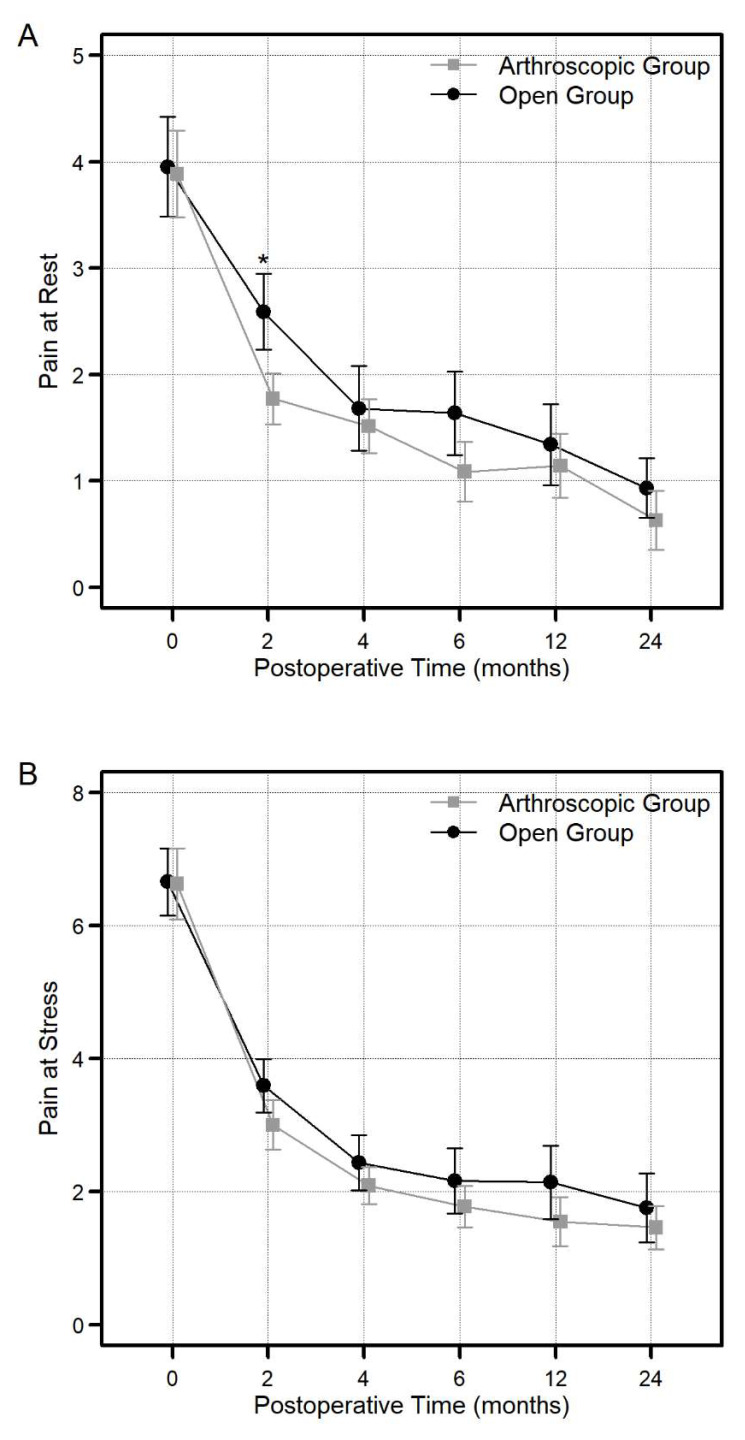
Comparison of the visual analog score (VAS) score at rest (**A**) and stress (**B**) between the arthroscopy and open groups at each postoperative timepoint (2, 4, 6, 12, and 24 months) (* significant difference, *p* < 0.05). The vertical bar graph represents the average along with the corresponding 95% confidence interval.

Both groups showed remarkable advancement in the mean follow-up VAS pain scores (at rest and at stress) 2 years after the operation, which were as follows: in the arthroscopy group, from 3.89 ± 1.18 preoperatively to 0.81 ± 0.81 at rest and from 6.63 ± 1.55 preoperatively to 1.46 ± 0.95 at rest (*p* < 0.001), and in the open group, from 3.95 ± 1.54 preoperatively to 0.93 ± 0.63 at rest and from 6.66 ± 1.67 preoperatively to 1.75 ± 1.71 at stress (*p* < 0.001). However, 2 years of postoperative follow-up VAS pain scores revealed no statistically significant difference between the two groups. Two months postoperatively, in terms of pain at rest, the average VAS score meaningfully improved in the arthroscopy group compared to the open group [1.77 ± 0.69 in the arthroscopy group vs. 2.59 ± 1.17 in the open group (*p* < 0.001)] (Figure 2A,B).

**Figure 3:**
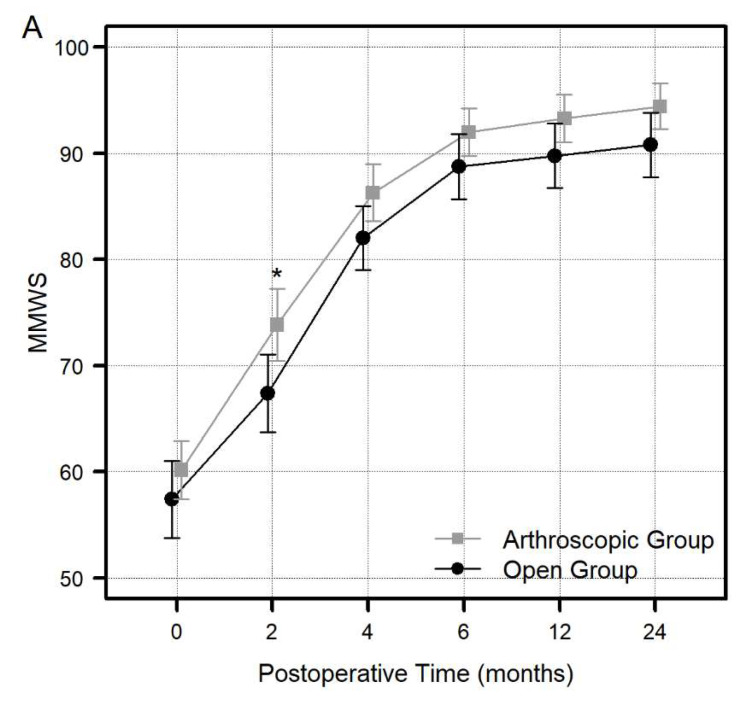

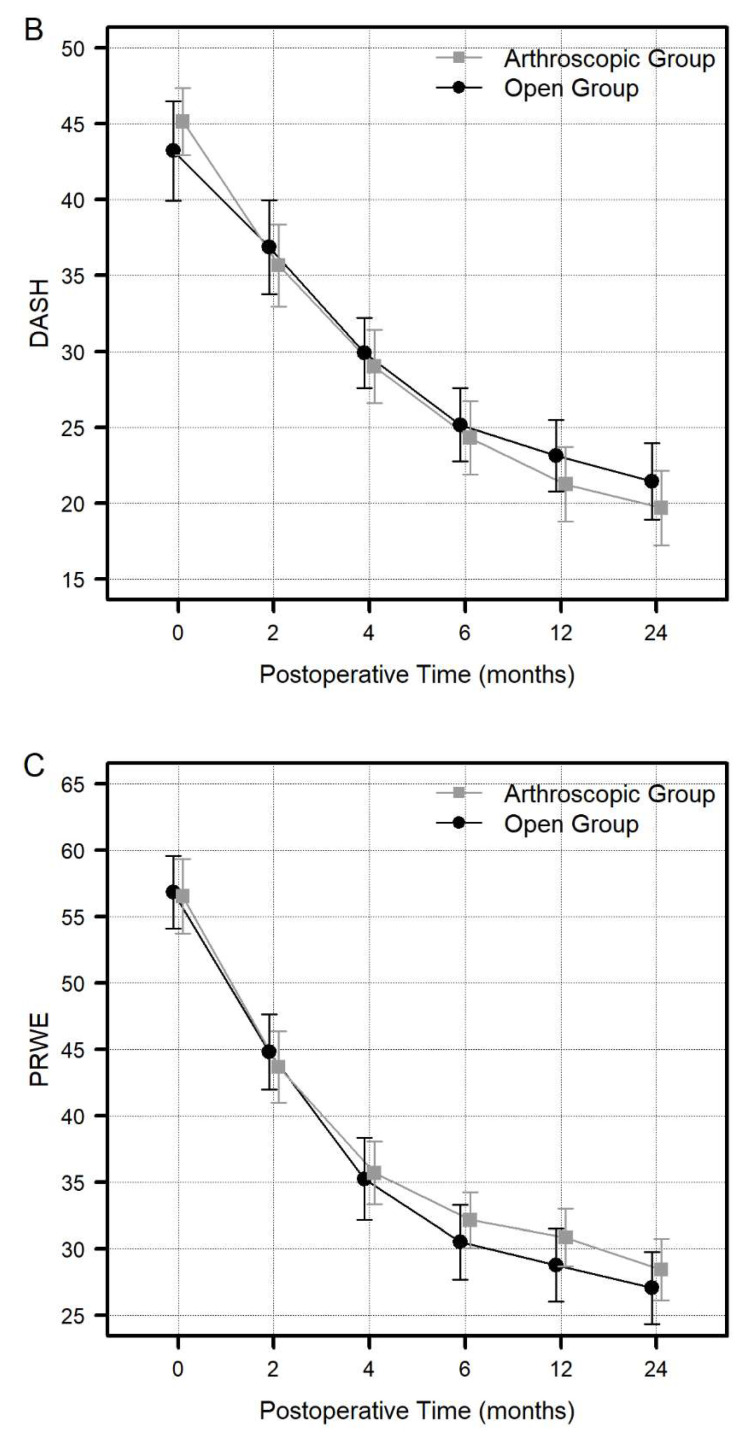
Comparison of the Mayo Modified Wrist Score (MMWS) (**A**), Disabilities of the Arm, Shoulder, and Hand (DASH) questionnaire scores (**B**), and the Patient-Rated Wrist Evaluation (PRWE) scores (**C**) between the arthroscopy and open groups at each specific postoperative timepoint (2, 4, 6, 12, and 24 months) (* *p*-value less than 0.05 were regarded as significant).

Two years postoperatively, we have discovered a notable increase in the MMWS (*p* < 0.001) and a decrease in the DASH (*p* < 0.001) and PRWE (*p* < 0.001) scores in both groups. However, in 2-year postoperative MMWS, the DASH and PRWE did not significantly differ between the arthroscopy and open groups (Table 2). Two months postoperatively, arthroscopy groups showed a significant difference in the MMWS scores relative to open repair groups (73.9 ± 9.9 points in the arthroscopic groups and 67.4 ± 12 points in the open group; *p* = 0.022); in contrast, two groups revealed no notable distinctions in the DASH (*p* = 0.56) and PRWE (*p* = 0.57) scores (Figure 3A–C).

### 3.4. ROM (Flexion and Extension Arc, Supination and Pronation Arc, Radial and Ulnar Deviation Arc)

**Figure 4:**
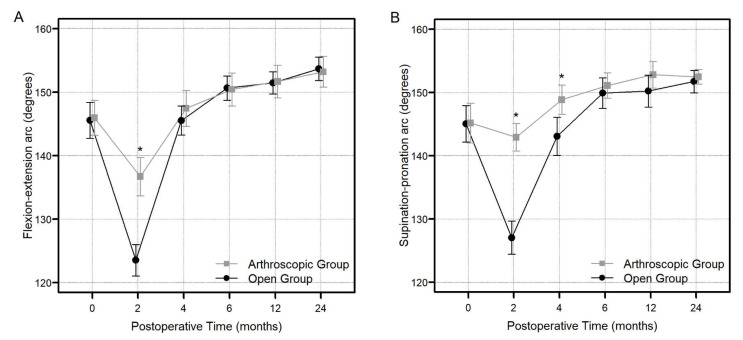
The comparison of the degree of flexion–extension arc (**A**) and supination–pronation arc of the forearm (**B**) between the arthroscopy and open groups at each specific postoperative timeline (2, 4, 6, 12, and 24 months) (* we regarded *p*-value less than 0.05 as significant).

The arthroscopy group showed a better flexion–extension arc at 2 months and supination–pronation arc at 2 and 4 months than the open group (*p* < 0.001) (Figure 4A,B).

However, authors observed no notable distinction in ROM amongst two groups at 2 years postoperatively [flexion–extension arc: 153.23 ± 7.06 in arthroscopy group and 153.70 ± 6.04 in open group (*p* = 0.944); supination–pronation arc: 152.49 ± 3.42 in arthroscopy group and 151.75 ± 5.83 in open group (*p* = 0.964); radial deviation–ulnar deviation arc: 52.37 ± 3.79 in arthroscopy group and 52.07 ± 4.44 in open group (*p* = 0.654)].

### 3.5. Grip Strength

At a 2-year follow-up, a significant increase was recognized in grip strength in both groups. (*p* < 0.001). Nevertheless, no statistically meaningful difference in grip power after surgery was found among arthroscopy and open groups (33.49 ± 9.84 vs. 34 ± 10.36; *p* = 0.82).

### 3.6. Complication Rates and Duration until Return-to-Work

**Figure 5:**
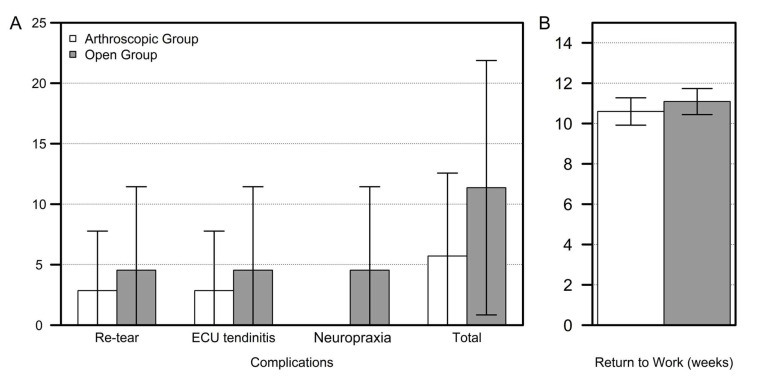
Comparison of complication rate (**A**) and return-to-work duration (**B**) between the arthroscopic and open groups.

We could not find any significant distinction in the rate of complications between the arthroscopy and open groups [11.4% (4 of 35) in the arthroscopy group and 18.2% (8 of 44) in the open group, *p* = 0.533; Figure 5A].

Recurrent DRUJ instability was identified in 7 (15.9%) of 44 participants in the open group and 3 (8.6%) among 35 patients in the arthroscopy group, with no notable distinction amongst the groups (*p* = 0.499). Of these, one patient in the arthroscopy group and two in the open group underwent severe trauma after the operation. The DRUJ ballottement test showed grades 1 and 2 instability in 1 and 2 patients, respectively, from the arthroscopic group and grades 3 and 2 in 2 and 3 patients, respectively, from the open group. We identified tendinitis of the ECU tendon after surgery in 3 (3.79%) of the total 79 patients (2 in the open group and 1 in the arthroscopy group). Two participants (4.5%) from the open group complained of neuropraxia, which involved the dorsal sensory branch of the ulnar nerve, with complete spontaneous restoration in 6 months. There were no wound infections or sensory changes in the affected limb. No patients complained of limitations in the range of wrist motion after surgery. We found no significant difference in return-to-work duration between the two groups (10.60 ± 1.97 vs. 11.09 ± 2.21 weeks; *p* = 0.423) (Figure 5B).

## 4. Discussion

Contrary to our hypothesis, we could not find any notable differences in the clinical results and complication rates among the arthroscopic and open repair techniques after surgery. In this study, those who experienced repairs of TFCC foveal injuries using either arthroscopic or open repair techniques had comparable postoperative clinical outcomes at a minimum 2-year follow-up. However, patients who underwent arthroscopic repair showed less postoperative pain and an earlier recovery of typical ROM.

Although open TFCC repair remained a primary treatment choice for TFCC foveal tear [23,30,31], the arthroscopic repair technique recently showed remarkable progress in treating TFCC foveal tear [14,30,32,33,34]. In the present study, the average DASH score decreased significantly from 45.17 preoperatively to 19.68 at 2 years after the operation in the arthroscopic repair group. We achieved resolution of DRUJ instability in 32 patients (91.4%), while 3 patients (8.6%) had recurrent DRUJ instability.

The arthroscopic-assisted repair technique, primarily presented by Atzei et al., is broadly utilized in TFCC foveal repair with a suture anchor [21,32]. Atzei et al. assessed the results involving 48 people who had gone all-inside arthroscopic technique using a suture anchor for the foveal repair of the TFCC, followed up for an average of 33 months. Unstable DRUJ has shown resolution in 44 patients, and the DASH score revealed significant improvement: preoperative average 42 ± 20 to postoperative average 15 ± 15 [15]. In 2011, Nakamura et al. revealed an outside-in arthroscopic procedure that created two distinct 1.2 mm trans-osseous passages through the radiocarpal joint utilizing a specifically designed device. We also evaluated the clinical results reported by 24 patients with a mean duration of 3.5 years of follow-up. A total of 15 patients (62.5%) did not complain of any pain, 2 patients (8.3%) had severe pain, and four patients (16.6%) had recurrent pain. Of these, 17 patients (70.8%) achieved complete resolution of DRUJ instability, while 7 patients (29.2%) reported a recurrence of DRUJ instability [33]. Shinohara et al. advanced Nakamura’s method by meticulously placing the trans-osseous tunnels into the fovea using arthroscopic visualization of DRUJ in place of the radiocarpal joint. We evaluated eleven cases for an average 30-month follow-up. Nine cases (81.8%) achieved resolution of DRUJ instability, whereas two cases (18.2%) had persisting minimal DRUJ instability [16]. However, clinical outcomes, such as MMWS, DASH, and PRWE scores, were not evaluated in this study.

There is growing evidence supporting successful outcomes of the TFCC and fovea repair in patients with unstable DRUJ [21,35]. However, there are still debates on the superiority of arthroscopic techniques to open repair techniques, which is a key question that remains to be answered [7,19]. In 2013, Luchetti et al. [36] introduced comparative research involving 49 people with foveal TFCC tears. Despite the lack of statistical distinction in ROM and VAS pain and the MMWS, DASH, and PRWE scores, the outcomes were significantly better with the arthroscopic procedure than with the open repair procedure. In addition, an increased rate of failure in the open repair technique group was observed [5/49 (10%) failure, 4 vs. 1]. However, this study included TFCC lesions associated with distal fractures (25/49, 51%), which affect wrist function per se and can act as a confounding factor. In addition, patients who were followed up for a minimal period of 6 months were also involved, making it difficult to confirm the midterm follow-up results.

In contrast, Abe et al. [37] introduced comparative clinical research that involved 29 TFCC-foveal tear patients. Despite a lack of significant distinctions between the open and arthroscopy groups, the DRUJ instability resolved in 19 out of 21 patients in the arthroscopy group and in 8 patients in the open group. However, the size of the samples in this study was relatively small, particularly for patients who had gone through the open repair technique.

In 2017, Ma et al. [18] researched the biomechanics of open TFCC repair with a suture anchor and arthroscopic trans-osseous repair of the TFCC foveal tear. The arthroscope-assisted suture technique through the bone tunnel reported superior stability to open repair with a suture anchor technique. In the present clinical study, three patients (8.6%) in the arthroscopic group and seven (15.6%) in the open group had recurrent DRUJ instability after surgery. Although not statistically significant, this may be attributed to type II error due to the relatively small sample size. There may be a need for additional studies for the evaluation of re-tear rates within different repair techniques.

Arthroscopic-assisted TFCC repair offers increased meticulous visual presentation of the TFCC tear site and confirmation of the integrity of sutured TFCC, thus leading to decreased risk of injury to the capsule and ECU sub-sheath, which are dynamic stabilizers of DRUJ [30]. Compared with the open repair approach, the arthroscopic approach is more advantageous because it is minimally invasive and less extensive, avoiding damage to the surrounding soft tissue anatomies [38]. Thereby, the incidence of postoperative adhesion is lower, and patients recover faster after arthroscopic repair than after open repair. In the present study, active ROM showed significant superiority after the arthroscopic repair over open repair in the early (2 and 4 months) postoperative months. Pain and MMWS at 2 months were also significantly improved in the arthroscopy group in comparison to those in the open group. However, the ROM of the arthroscopy group became comparable to the open group 6 months after the operation, and no notable distinction in return-to-work duration was found between both groups.

### Limitations

Our study has several limitations. First, it was conducted retrospectively; thus, patients were not assigned randomly to the treatment groups, and we cannot preclude the occurrence of selection bias. Second, we included a comparatively small sample size, which might elevate the risk of committing type II statistical error when comparing outcome measures like complication rate or patient demographics. Third, we did not analyze whether accompanying procedures (for instance, a wafer procedure or an ulnar shortening osteotomy) had an effect on the clinical outcome. Nevertheless, we found no meaningful distinction in the incidence of ulnar impaction syndrome between the arthroscopic and open groups. Thus, it was assumed that the accompanying procedure would not affect the result between the arthroscopic and open groups. Further research could be conducted to evaluate the effect of accompanying procedures. Fourth, we did not systematically assess the postoperative condition of TFCC through postoperative MRI or second-look arthroscopy. Therefore, the objective of the TFCC healing status could not be determined from this investigation. However, the strength of this study is that physical examination, for instance, the ballottement test, and data were collected by two qualified orthopaedists, S.L. and J.H., which is anticipated to diminish the observer bias.

## 5. Conclusions

Patients who underwent repair of a TFCC foveal tear with either arthroscopic or open repair had similar clinical outcomes at a minimum 2-year follow-up. Compared with the open repair technique, arthroscopic repair of a TFCC foveal tear provided early restoration of range of motion and pain through a minimally invasive technique.

## Figures and Tables

**Table 1:** Patient characteristics in the arthroscopy and open groups.

Demographic Data	Arthroscopy Group (n = 35)	Open Group (n = 44)	*p*-Value
Age (years, mean ± standard deviation)	40.4 ± 12.7	38.9 ± 14.0	0.637
Sex (F:M)	15:20	19:25	0.999
Affected side (right:left)	20:15	21:23	0.545
Smoking history (yes:no)	26:9	30:14	0.731
Time to surgery, months, mean ± standard deviation	15.7 ± 21	11.7 ± 11.3	0.340
Concomitant ulnar impaction syndrome, n	11	12	0.877

**Table 2:** Table comparing clinical outcomes.

	Arthroscopic Group (n = 35)	Open Group (n = 44)	*p*-Value
VAS at rest			
Preoperative	3.89 ± 1.18	3.95 ± 1.54	0.869
2-year follow-up	0.63 ± 0.81	0.93 ± 0.93	0.115
VAS at stress			
Preoperative	6.63 ± 1.55	6.66 ± 1.67	0.899
2-year follow-up	1.46 ± 0.95	1.75 ± 1.71	0.756
MMWS			
Preoperative	60.14 ± 8.0	57.39 ± 11.98	0.441
2-year follow-up	94.43 ± 6.27	92.80 ± 9.94	0.095
DASH			
Preoperative	45.17 ± 6.46	43.23 ± 10.80	0.234
2-year follow-up	19.68 ± 7.18	21.42 ± 8.26	0.570
PRWE			
Preoperative	56.54 ± 8.19	56.86 ± 9.03	0.870
2-year follow-up	28.43 ± 6.71	27.05 ± 8.97	0.450

Values are given as mean ± standard deviation. VAS, visual analog scale; MMWS, Mayo Modified Wrist Score; DASH, Disabilities of the Arm, Shoulder, and Hand questionnaire score; PRWE, Patient-Rated Wrist Evaluation score.

## Data Availability

The data presented in this study are available on request from the corresponding author.

## Supplementary Materials

The following supporting information can be downloaded at: https://www.mdpi.com/article/10.3390/jcm13102766/s1, Video S1: Arthroscopic foveal repair of the TFCC. Video S2: Open foveal repair of the TFCC.
